# MST1/Drp1 axis mediates microglia pro-inflammatory activation following cerebral ischemia-reperfusion injury

**DOI:** 10.1038/s41598-026-57741-6

**Published:** 2026-06-13

**Authors:** Yao Mu, Jianping Liu, Yi Dong, Meihan Li, Yang Yuan, Bowen Wang, Wenjie Liu, Bingqiang Zhang, Rui Dong, Xuehua Sun, Gaofeng Zhang

**Affiliations:** 1 The Second School of Clinical Medicine of Binzhou Medical University, Yantai, 264000 Shandong China; 2 Department of Anesthesiology, Qingdao Hospital, University of Health and Rehabilitation Sciences (Qingdao Municipal Hospital), Qingdao, 266071 Shandong China; 3 Doctor-Patient Communication Office, Qingdao Hospital, University of Health and Rehabilitation Sciences (Qingdao Municipal Hospital), Qingdao, 266071 Shandong China; 4 School of Anesthesiology, Shandong Second Medical University, Weifang, 261053 Shandong China; 5 Research Institute, Qingdao Restore Biotechnology Co., Ltd., Qingdao, 266071 Shandong China; 6 Department of Pain Medicine, Yantai Affiliated Hospital of Binzhou Medical University, Yantai, 264000 Shandong China

**Keywords:** Cerebral ischemia-reperfusion injury, Microglia, Microglia pro-inflammatory activation, Mammalian sterile 20-like kinase 1, Dynamin-related protein 1, Mitochondrial fission, Cell biology, Neurology, Neuroscience

## Abstract

The present study investigated the role of the mammalian sterile 20-like kinase 1/dynamin-related protein 1 (MST1/Drp1) axis in regulating microglia pro-inflammatory activation during cerebral ischemia-reperfusion injury (CIRI). An in vivo model of middle cerebral artery occlusion/reperfusion (MCAO/R) in rats and an in vitro oxygen-glucose deprivation/reoxygenation (OGD/R) model in BV-2 microglial cells and primary microglia were established. Inhibitors of MST1 (XMU-MP-1) or/and Drp1 (Mdivi-1), along with genetic approaches including siMST1-mediated knockdown and plasmid-based overexpression, were utilized in the models. The expression and activation of MST1 and Drp1, mitochondrial morphology changes, microglia pro-inflammatory activation makers, pro-inflammatory cytokine release, DNA fragmentation and neurological function were evaluated. The findings indicated that reperfusion or reoxygenation led to a rise in total and phosphorylation levels of MST1 and Drp1. The reperfusion also facilitated the Drp1 translocation toward mitochondria, and resulted in increased mitochondrial morphological changes. MST1 or/and Drp1 inhibitors decreased p-MST1 and p-Drp1(Ser616) levels, attenuated mitochondrial fission, suppressed microglia pro-inflammatory activation, pro-inflammatory factors release (TNF-α, IL-6 and IL-1β). Overall, these effects ultimately mitigated cerebral injury as evidenced by reduced DNA fragmentation, decreased cerebral infarct volumes, and improved neurological function. Combined inhibitors further exerted ameliorative effects on the above-mentioned parameters. In the in vitro experiments, siMST1 knockdown attenuated p-Drp1(Ser616) expression and suppressed microglia pro-inflammatory activation under OGD/R conditions. These protective effects were reversed by Drp1 overexpression. These findings indicate that p-MST1 drives microglia pro-inflammatory activation via promoting the p-Drp1(Ser616)-mediated excessive mitochondrial fission during CIRI.

**Supplementary Information:**

The online version contains supplementary material available at https://doi.org/10.1038/s41598-026-57741-6.

## Introduction

Cerebral ischemia-reperfusion injury (CIRI) describes the occurrence where the reestablishment of blood flow after ischemia unexpectedly worsens initial brain damage^1^. Study suggests that CIRI involves multiple signaling pathways, with its mechanisms potentially linked to mitochondrial dysfunction, intracellular calcium overload, DNA fragmentation, inflammatory damage, along with additional factors^2,3^. In the central nervous system (CNS), microglia are the key immune regulators during CIRI. Microglia can be overactivated toward a pro-inflammatory profile in response to ischemic reperfusion insult, thereby exacerbating brain damage by releasing abundant pro-inflammatory mediators during the acute phase of CIRI^4,5^.

Mitochondria undergo constant cycles of fusion and fission. This shifting balance preserves the morphology and function to adapt to cellular metabolic and stress requirements^6^. One of the key characteristics of CIRI is the excessive mitochondrial fission, which is driven by the action of dynamin-related protein 1 (Drp1)^7^. After phosphorylation at Ser616 residue (p-Drp1(Ser616)), Drp1 translocate from cytosol to mitochondrial outer membrane. This translocation triggers excessive mitochondrial fission, which exacerbates neuronal injury^8^. Studies further found that in both lipopolysaccharide (LPS) stimulation model and experimental autoimmune myocarditis (EAM) model, p-Drp1(Ser616) and the subsequent mitochondrial fission increase reactive oxygen species (ROS) generation, ultimately promotes pro-inflammatory activation of microglia/macrophages, and induces the release of pro-inflammatory factors^9,10^.

Mammalian sterile 20-like kinase 1 (MST1) is a crucial element in Hippo pathway and significantly contributes to responses related to oxidative stress and inflammation^11,12^. Notably, in subarachnoid hemorrhage model, Qu et al. demonstrated that MST1 triggers the NF-κB/MMP-9 pathway, which is implicated in driving the secretion of pro-inflammatory mediators. This cascade ultimately leads to the disruption of the blood–brain barrier (BBB) and exacerbates brain edema^13^. In studies involving myocardial infarction (MI) and stimulation by lipopolysaccharide (LPS), it has been demonstrated that MST1 promotes the phosphorylation of the downstream target JNK, which in turn activates mitochondrial fission mediated by Drp1^14,15^.

Currently, the potential impact of MST1 on the microglia pro-inflammatory activation through modulating Drp1 phosphorylation during CIRI has yet to be clarified. This research aims to discover the possible mechanisms by which the MST1/Drp1 axis regulate microglia pro-inflammatory activation in CIRI using a MCAO/R model and an OGD/R model.

## Materials and methods

### Animals

Male Sprague-Dawley rats classified as specific-pathogen-free (SPF), aged between 6 and 8 weeks and weighing between 240 and 260 g, were bought from Shandong Pengyue Laboratory Animal Technology Co., Ltd. (Quality license No. SCXK(Lu)20220006). These animals were maintained in a regulated environment with a temperature of 20 to 24 °C, relative humidity of 50 to 60%, and a light/dark cycle of 12 h, with free access to standard food and water. The Ethics Committee Medical College of Qingdao University granted approval for the study protocol (approval No. QDU-AEC-2024473). All experimental procedures were conducted in accordance with the National Institutes of Health Guide for the Care and Use of Laboratory Animals (NIH publication 23–80, revised 2011). All experiments involving animals are documented according to ARRIVE guidelines^16^.

All rats were randomly divided into five groups (*n* = 25/group): Sham group (S group): only exposure of cervical vessels without occlusion; Ischemia/reperfusion group (I/R group): occlusion of the middle cerebral artery for 2 h followed by reperfusion; MST1 inhibitor group (IM group): administration of XMU-MP-1 before MCAO; Drp1 inhibitor group (ID group): administration of Mdivi-1 before MCAO; Combined inhibitors group (IMD group): administrations of both XMU-MP-1 and Mdivi-1 before MCAO.

### Creation of the CIRI model

A CIRI model was established using the intraluminal filament occlusion method^17^. Rats were anesthetized by intraperitoneal (i.p.) administration of 1% pentobarbital sodium (40 mg/kg) and subsequently fixed in a temperature-controlled surgical table. Following disinfection, a midline incision was made on the neck to expose the common carotid artery (CCA), external carotid artery (ECA), and internal carotid artery (ICA). A suture was threaded through ECA into the ICA. Its tip was placed approximately 20 mm past the bifurcation of the CCA to block the middle cerebral artery. The suture was then gently removed after the two-hour occlusion period, thus allowing blood flow to resume and reperfusion to occur. Cerebral blood flow (CBF) was monitored using laser Doppler flowmetry. Only animals demonstrating a reduction in cerebral blood flow (CBF) greater than 80% during the occlusion phase, along with a post-reperfusion recovery of over 80%, were included in the study^18^. Animals in the sham group underwent identical surgical processes yet did not experience arterial occlusion.

### BV-2 cells and primary microglia OGD/R model and group

BV-2 microglial cells were purchased from iCell Bioscience Inc. (Shanghai, China). Primary microglia were isolated from the cerebral cortex of 1-2-day-old neonatal SD rats^19^. Briefly, cortical tissues were minced, digested with 0.25% trypsin-EDTA, and filtered/centrifuged to prepare single-cell suspensions. Cells were seeded onto poly-D-lysine-coated flasks in DMEM (10% FBS, 1% penicillin-streptomycin) and cultured at 37 °C with 5% CO₂ (medium changed every 2–3 days). After 10–14 days, microglia were purified by orbital shaking (200 rpm, 2 h) and replated. BV-2 cells and primary microglia were maintained in high-glucose DMEM (10% FBS, 1% penicillin-streptomycin) under the same incubator conditions. The OGD/R model was established as reported^20^: cells were incubated in glucose-free DMEM under hypoxia (1% O₂, 5% CO₂, 94% N₂) for 6 h, followed by 24 h reoxygenation in high-glucose DMEM to mimic I/R injury. BV-2 cells were divided into five groups: Control group (C group); OGD/R group; MST1 inhibitor group (OM group): pretreatment with XMU-MP-1 before OGD; Drp1 inhibitor group (OD group): pretreatment with Mdivi-1 before OGD; Combined inhibitor group (OMD group): pretreatment with both XMU-MP-1 and Mdivi-1 before OGD. Primary microglia were divided into four groups: control + siNC + empty vector (C + siNC + Vector), OGD/R + siNC + Vector, OGD/R + siMST1 + Vector, and OGD/R + siMST1 + Drp1 OE.

### Intervention in animals and cells

For the in vivo experiments, the inhibitors XMU-MP-1 (A397171, Ambeed, USA) and Mdivi-1 (A252480, Ambeed, USA) were prepared by dissolving and diluting them in a solution that included 10% DMSO, 40% PEG 300, 5% Tween-80, and 45% saline. Based on prior research, XMU-MP-1^21^ was given through i.p. at a dosage of 4 mg/kg on alternate days over 5 days before the induction of MCAO in IM group and IMD group. Mdivi-1^22^ was given through i.p. at a dosage of 20 mg/kg one hour before induction of MCAO in ID group and IMD group.

For the in vitro experiments of BV-2 cells, the inhibitors XMU-MP-1 and Mdivi-1 were solubilized in DMSO, with final working concentrations of 5 µM and 20 µM, respectively. Based on prior research, XMU-MP-1^21^ were added to the culture medium 30 minutes prior to OGD exposure in OM group and OMD group. Mdivi-1^23^ were added to the culture medium 30 minutes prior to OGD exposure in OD group and OMD group. For the primary microglia, the cells were transfected with siRNA or plasmid 2–3 days after being seeded into a 12-well plate. The siRNA and pcDNA3.1-Drp1 were purchased from GenePharma (Shanghai, China) and Cyagen Company (Guangzhou, China), respectively. For siRNA-mediated knockdown of MST1, cells at 60–80% confluence were first transfected with 30 nM siMST1 or negative control siRNA (siNC) according to the manufacturer’s instructions. The siRNA sequence used to silence MST1 was as follows: 5’-GCAGGUCAACUUACAGAUATT-3’. For Drp1 overexpression, primary microglia were transfected with either 2 µg pcDNA3.1-Drp1 overexpression plasmid (Drp1 OE) or empty vector (pcDNA3.1) according to the manufacturer’s instructions. Knockdown and overexpression efficiencies were assessed 48 h after siRNA transfection and plasmid transfection by Western blot. After 48 h of transfection, the cells were subjected to OGD/R as described before.

### Neurological function deficit evaluation

The modified neurological severity score (mNSS) is a comprehensive neurobehavioral assessment tool designed to evaluate neurological deficits^24^. This scoring system quantifies four core domains of neurological function: motor performance, sensory function, reflex integrity, and motor coordination/balance. The total score spans from 0 to 18 points, where the 0 means no neurological impairment and the 18 represents severe neurological deficits. Increased scores suggest more significant neurological damage. The assessment was performed at 24 h after reperfusion for all rats. After mNSS, the rats were euthanized by intraperitoneal injection of pentobarbital sodium (150 mg/kg), and then the following tests were performed.

### Immunofluorescence staining

After the mNSS evaluation, five rats were randomly chosen, anesthetized deeply, and underwent intracardiac perfusion with PBS. This was subsequently followed by perfusion with 4% paraformaldehyde. The ischemic penumbra region (the junction of ischemic necrotic and normal tissue) was dissected and preserved in 4% paraformaldehyde at 4 °C for a duration of 24 h. Paraffin-embedded brain sections (5 μm) were first deparaffinized and subjected to antigen retrieval, with endogenous enzymes also blocked. Subsequently, they were treated with 10% goat serum for 30 min at room temperature. Finally, an overnight incubation at 4 °C with primary antibodies was carried out. The primary antibodies: anti-iNOS (18985-1-AP, 1:200, Proteintech), anti-CD86 (CY5238, 1:200, Abways), anti-Iba-1 (CY7217, 1:200, Abways), anti-MST1 anti-MST1(66663-1-Ig, 1:200, Proteintech), anti-P-MST1(#49332, 1:200, CST), anti-Drp1(12957-1-AP, 1:200, Proteintech), anti-p-Drp1(Ser616) (#3455, 1:200, CST), and anti-p-Drp1(Ser637) (#4867, 1:1000, CST). After washing, secondary antibodies (goat anti-rabbit, ab150080, 1:500, Abcam; goat anti-mouse, ab150113, 1:500, Abcam) were added and incubated at room temperature for one hour. Nuclei were then counterstained using DAPI (P0131, 1:1000, Beyotime). Fluorescence microscopy (Leica Microsystems, Wetzlar, Germany) was utilized to record the images. Random selections were made, including five sections for each group and five microscopic fields within each section. Subsequently, the CD86 or iNOS positive area within the Iba-1⁺ area was quantified and normalized to the total Iba-1⁺ area, and the other protein fluorescence intensity of whole field-of-view was measured and normalized to DAPI using ImageJ software^25^.

### Western blot analysis

Following a 24-hour reoxygenation period, cells were collected and washed with PBS. Protein extraction was performed utilizing RIPA lysis buffer that was enriched with protease and phosphatase inhibitors. Cytosolic and mitochondrial proteins were isolated through differential centrifugation for the subsequent detection of Drp1 on the mitochondrial membrane(M-Drp1)^26^. COX-Ⅳ served as loading controls for the mitochondrial, and β-actin was used to verify the purity of mitochondrial isolation. Protein levels were assessed using the BCA method. Equal protein amounts (30 µg for each lane) were subjected to 10% SDS-PAGE and subsequently transferred onto PVDF membranes. Membranes were initially treated for 1.5 h with 5% non-fat milk for blocking. Following this, the membranes underwent incubation at 4 °C overnight with a selection of primary antibodies: anti-iNOS (PA1-036, 1:1000, Invitrogen), anti-CD86 (MA5-32078, 1:10000, Invitrogen), anti-MST1 (66663-1-Ig, 1:5000, Proteintech), anti-p-MST1 (#49332, 1:1000, CST), anti-Drp1 (12957-1-AP, 1:5000, Proteintech), anti-p-Drp1(Ser616) (#3455, 1:1000, CST), anti-p-Drp1(Ser637) (#4867, 1:1000, CST), anti-COX Ⅳ (DF5468, 1:1000, Affinity), and anti-β-actin (66009-1-Ig, 1:3000, Proteintech). After washing, the membranes underwent treatment with secondary antibodies conjugated with horseradish peroxidase anti-mouse or anti-rabbit (#7076,1:2000; #7074, 1:2000, CST) for 1.5 h. Immunoreactive bands were identified through the application of an enhanced chemiluminescence (ECL) reagent (Invitrogen). Band were analyzed using ImageJ software. The results were presented as the mean values obtained from three separate replicates.

### Transmission electron microscopy (TEM)

After mNSS, sodium pentobarbital was used to anesthetize 5 rats. For fixation, intracardiac perfusion was performed sequentially with saline and 2.5% glutaraldehyde. The brains were then immediately dissected. The ischemic penumbra region was obtained and cut into 1 mm³ cubes. Following initial fixation in 2.5% glutaraldehyde for one day, the samples were post-fixed with 1% osmium tetroxide for two-hour. They were then dehydrated in an ascending acetone series prior to resin embedding. Ultrathin slices (70 nm) were generated and stained twice using uranyl acetate and lead citrate. JEM1400F microscope (JEOL, Tokyo, Japan) was used and representative images were recorded. A randomized sampling approach was employed, selecting 5 sections for each animal group and 5 microscopic fields within each section. Mitochondrial aspect ratio and the percentage of swollen mitochondria were quantified using ImageJ software^27,28^.

### Mitochondrial Drp1 localization under confocal imaging

Laser Confocal Scanning imaging was used to determine the localization of Drp1 to mitochondria^29^. BV-2 cells were plated on sterile glass coverslips and cultured until reaching 70–80% confluence, followed by OGD/R modeling as described above and staining. Mitochondria were labeled by incubation with 100nM TMRM (T668, Invitrogen) at 37℃ for thirty minutes, protected from light. Subsequently, they were washed gently and fixed using 4% paraformaldehyde for fifty minutes. After that, BV-2 cells were permeabilized using 0.1% Triton X-100 for 10 min and blocked with BSA for 1 h. Next the cells were probed overnight with anti-Drp1 primary antibody (ab184247,1:500, Abcam). After that, cells were treated with the secondary antibody conjugated to Alexa Fluor 488 (A-11008,1:500, Invitrogen) under dark condition, rewashed, and counterstained with 1 µg/mL DAPI for nuclear visualization. Fluorescence pictures were recorded by a LSM 900 confocal laser scanning microscope (Zeiss, Oberkochen, Germany) with the following excitation/emission wavelengths: 490/510 nm for Drp1-Alexa Fluor 488, 410/450 nm for DAPI, and 550/600 nm for TMRM. M-Drp1 was defined as the fraction of Drp1 signal (green) co-localizing with mitochondria (red). T-Drp1 was defined as the cellular Drp1 signal (green). ImageJ software was used for colocalization analysis. The M-Drp1/T-Drp1 ratio was calculated as the fluorescence intensity of M-Drp1 divided by that of T-Drp1. Three independent coverslips per group were assayed, and five random fields per coverslip were quantified for data collection.

### Pro-inflammatory cytokines production assay in vivo and in vitro

The brain tissue was homogenized ultrasonically on ice for 30 min. Subsequently, the homogenized tissue was centrifuged at 4 °C and 12,000 × g for 10 min, and the supernatant was collected. For cell culture samples, the BV-2 cells and primary microglia were subjected to 24 h of reoxygenation, after which the culture supernatant was collected and centrifuged at 1,000 × g for 5 min at 4 °C to remove debris. The secretion of IL-6, TNF-α and IL-1β were assessed using enzyme-linked immunosorbent assay (ELISA) kits (Beyotime, China), in line with the guidelines. Measurements were taken at 450 nm with a microplate reader. The level of each cytokine was assessed by comparing it to the standard curve.

### HE staining and TUNEL immunofluorescence staining

Ten rats were randomly chosen after mNSS, 5 for HE staining and 5 for TUNEL immunofluorescence staining. The anesthetized rats were subjected to heart perfusion and the brain tissue was preserved using 4% paraformaldehyde. Subsequently, the tissue samples that had been fixed were typically dehydrated, clarified, and embedded in paraffin. To perform HE staining, the brain tissue slices underwent deparaffinization and rehydration. They were initially stained with hematoxylin to visualize the nuclei, followed by a differentiation and bluing process, and subsequently stained with eosin to highlight the cytoplasm. Following dehydration, the sections were photographed using an Olympus microscope (400×magnification). For morphological analysis, five randomly selected high-power fields (HPF) were captured from each of five sections.

For TUNEL immunofluorescence staining, the paraffin sections underwent deparaffinization and rehydration as previously outlined. Subsequently, the sections were exposed to proteinase K (20 µg/mL) at room temperature for a duration of 15 min. The TUNEL assay (Meilunbio, China) was conducted according to the description. The samples were treated with the TdT/dUTP labeling mixture for one hour and subsequently stained with DAPI to visualize nuclei. Fluorescence microscopy (Leica, Wetzlar, Germany) was utilized to capture images. TUNEL/DAPI double-positive cells were defined as cells with DNA fragmentation^30^. The rate was determined by counting TUNEL-positive cells within five randomly selected fields across each of five sections, employing the formula: TUNEL-positive cells Rate (%) = (Number of TUNEL-positive cells/Total cell count) × 100%.

### Cerebral infarction volume measurement

After mNSS, 5 rats were randomly selected. Following euthanasia, the brains were removed and stored at -20 °C for twenty minutes. Coronal sections, each measuring 2 mm in thickness, were stained using 2% 2,3,5-triphenyltetrazolium chloride (TTC, Sigma Aldrich, Burlington, MA, USA) in darkness for 20 min. The sections were fixed with 4% paraformaldehyde. Normal tissue was observed to be red, in contrast to the pale appearance of infarcted tissue. The volume of the infarct was determined using ImageJ software, employing the formula: Infarct volume(%)=(Contralateral hemisphere volume-Non-infarcted ipsilateral hemisphere volume)/Contralateral hemisphere volume×100%.

### Statistical analysis

All data analyses were conducted utilizing SPSS software 27.0 and GraphPad Prism software 10.0. The normality was assessed through the Shapiro-Wilk test. Results for data that followed a normal distribution are expressed as mean ± standard deviation (mean ± SD). To compare the differences among groups, one-way analysis of variance (ANOVA) with Tukey’s post hoc test was performed. For non-normally distributed data, results are presented as median with interquartile range (IQR) and are compared using the Kruskal-Wallis test with Dunn’s post hoc test. A significance level of p-value less than 0.05 was deemed statistically significant.

To minimize subjective bias, all quantitative assessments (including data collection, image acquisition, histological examinations, and statistical analysis) were conducted by investigators who were blinded to the experimental group assignments. No investigator involved in data acquisition or analysis had access to information regarding the grouping of individual samples or animals.

## Results

### CIRI increases the level of MST1 and promotes its phosphorylation

**Fig. 1:**
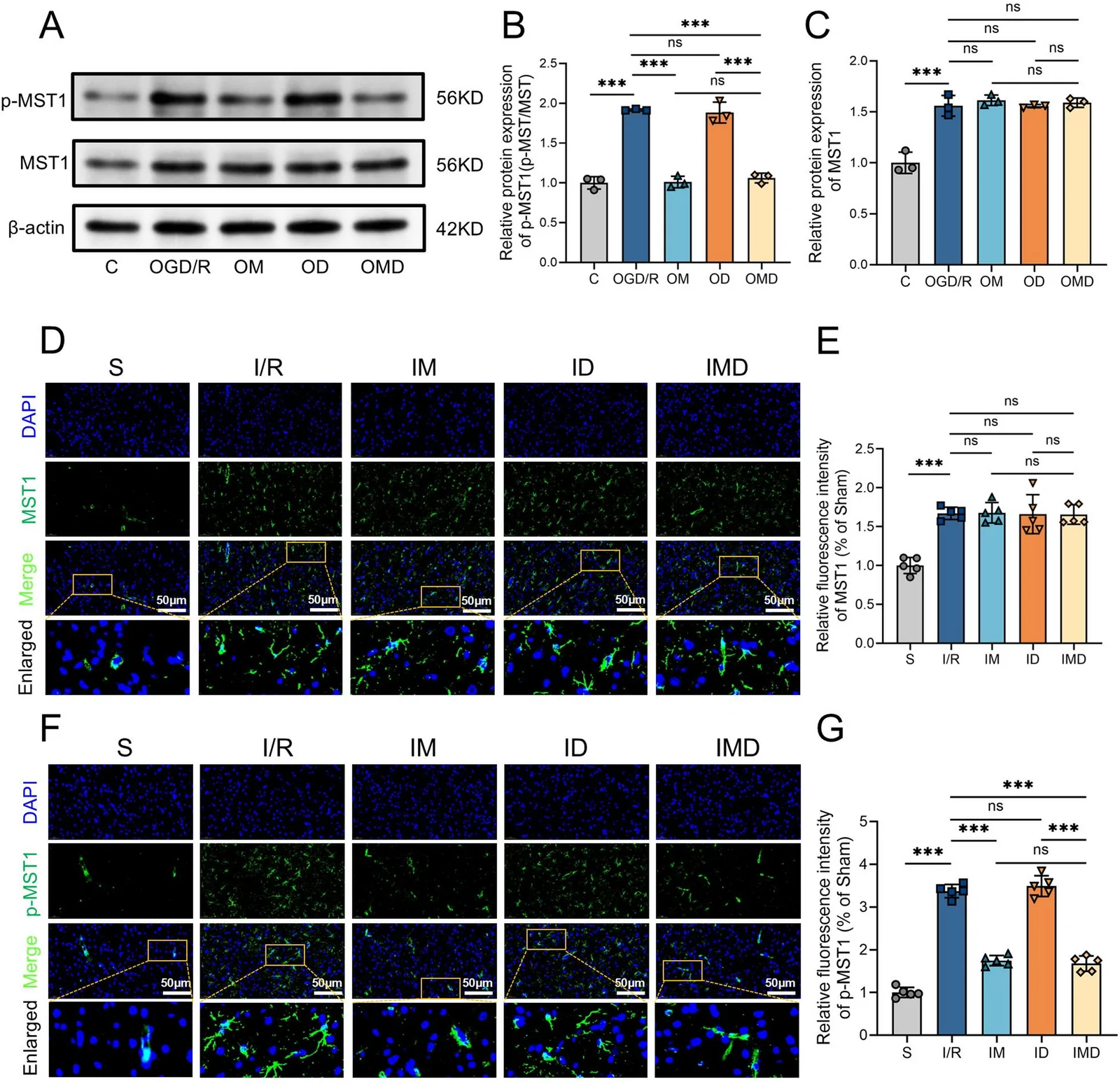
Levels of MST1 and p-MST1 increased in OGD/R-treated BV-2 cells as well as in brain tissues of I/R rats. (**A**) Representative Western blot bands showing the protein expression levels of MST1 and p-MST1 in BV-2 cells. MST1 and β-actin was used as a loading control. (**B**,**C**) Densitometric quantification of relative p-MST1 (**B**) and MST1 (**C**) proteins levels normalized to MST1 and β-actin, respectively (*n* = 3). (**D**,**F**) Representative immunofluorescence images of MST1 and p-MST1 (green) in the ischemic penumbra. Nuclei were counterstained with DAPI (blue). (**E**,**G**) The bar graphs represent the quantification of MST1 (**E**) or p-MST1 (**G**) fluorescence intensity, normalized to DAPI (*n* = 5). Scale bar: 50 μm. Data are expressed as mean ± SD. Statistical analysis was conducted via one-way ANOVA followed by Tukey’s post hoc test. ***p < 0.001; ns, no significant, *p*>0.05.

Western blot assays revealed that the amounts of MST1 and p-MST1 were notably elevated in the other four groups in comparison to the C group (Fig. 1A-C). Notably, administration of the MST1 inhibitor XMU-MP-1 to the OM and OMD groups reduced p-MST1 levels following OGD/R, and the inhibitory effects showed no difference between the two groups. The Drp1 inhibitor Mdivi-1 in the ID group barely exhibited any suppressive effect on p-MST1 levels. Neither of the two inhibitors showed a notable impact on MST1 levels compared with the OGD/R group.

Immunofluorescence staining exhibited similar results. There was a substantial increase in the relative intensity of MST1 and p-MST1 in the ischemic penumbra tissue among the four groups following perfusion compared with S group. However, no obvious difference was detected in the relative intensity of MST1 among the four reperfusion groups. In the IM and IMD groups, the expression of p-MST1 was reduced after the inhibition of MST1. There was no change on the relative fluo-intensity of p-MST1between I/R and ID group, or between IM and IMD groups (Fig. 1D-G). The outcomes indicate that Mdivi-1 has no effects on the p-MST1 level.

### P-MST1 promotes the level of p-Drp1(Ser616) during CIRI

**Fig. 2:**
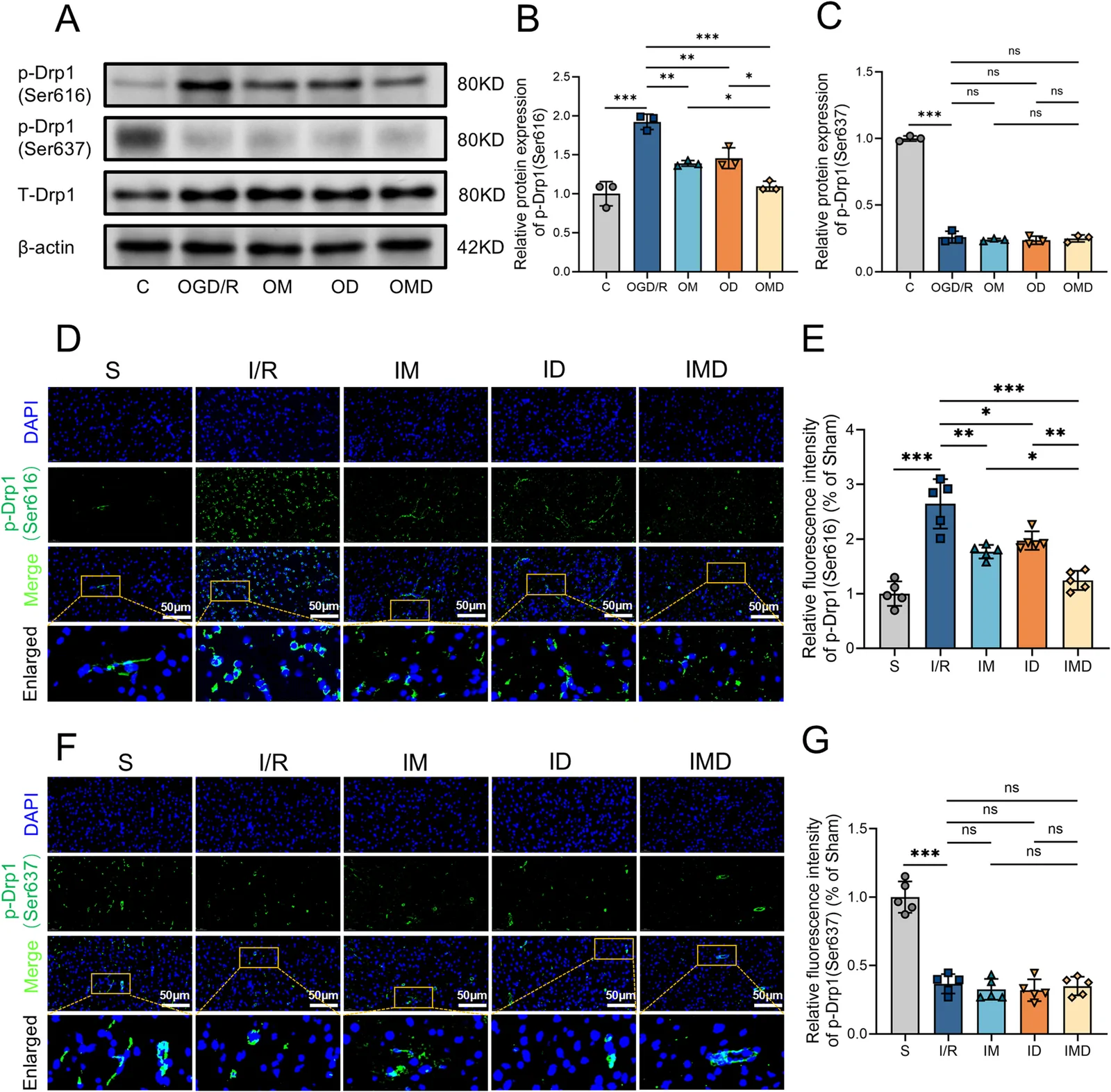
MST1 phosphorylation promotes the level of p-Drp1(Ser616) during CIRI. (**A**) Representative Western blot bands showing the protein expression levels of p-Drp1(Ser616) and p-Drp1(Ser637) in BV-2 cells. T-Drp1 was used as a loading control. (**B**,**C**) Densitometric quantification of relative p-Drp1(Ser616) (**B**) and p-Drp1(Ser637) (**C**) proteins levels normalized to T-Drp1 (*n* = 3). (**D**,**F**) Representative immunofluorescence images of p-Drp1(Ser616) and p-Drp1(Ser637) (green) in the ischemic penumbra. Nuclei were counterstained with DAPI (blue). (**E**,**G**) The bar graphs represent the quantification of p-Drp1(Ser616) (**E**) or p-Drp1(Ser637) (**G**) fluorescence intensity, normalized to DAPI (*n* = 5). Scale bar: 50 μm. Data are expressed as mean ± SD. Statistical analysis was conducted via one-way ANOVA followed by Tukey’s post hoc test. *p < 0.05, **p < 0.01, ***p < 0.001; ns, no significant, *p*>0.05.

The analysis performed via Western blot on BV-2 cells that underwent OGD/R demonstrated a significant rise in the levels of p-Drp1(Ser616) in comparison to the C group. Conversely, the levels of p-Drp1(Ser637) declined after OGD/R treatment (Fig. 2A-C). The application of either XMU-MP-1 or Mdivi-1 resulted in a reduction of p-Drp1(Ser616) levels. Notably, combined inhibitor treatment further downregulated the p-Drp1(Ser616) level relative to the OM or OD group. However, treatment with XMU-MP-1, Mdivi-1, or a combination of both had no effect on the p-Drp1(Ser637) levels.

Consistent with these findings, immunofluorescence staining displayed that the relative intensity of p-Drp1(Ser616) in the ischemic penumbra was increased to varying degrees after perfusion. The expression of p-Drp1(Ser616) is reduced by treatment with either MST1 inhibition or Drp1 inhibition, with the combined treatment (IMD group) producing a greater reduction (Fig. 2D, E). In line with the Western blot results, none of the inhibitor treatments changed the relative intensity of p-Drp1(Ser637) when compared with the I/R group (Fig. 2F, G). Collectively, these demonstrate that the MST1 inhibitor exhibited a similar effect to the Drp1 inhibitor on p-Drp1(Ser616). This suggests that MST1 activation regulates the level of p-Drp1(Ser616) during CIRI. In addition, both MST1 and Drp1 inhibitors selectively suppressed p-Drp1(Ser616) rather than p-Drp1(Ser637).

### P-MST1 exacerbates mitochondrial morphological changes through Drp1 activation

**Fig. 3:**
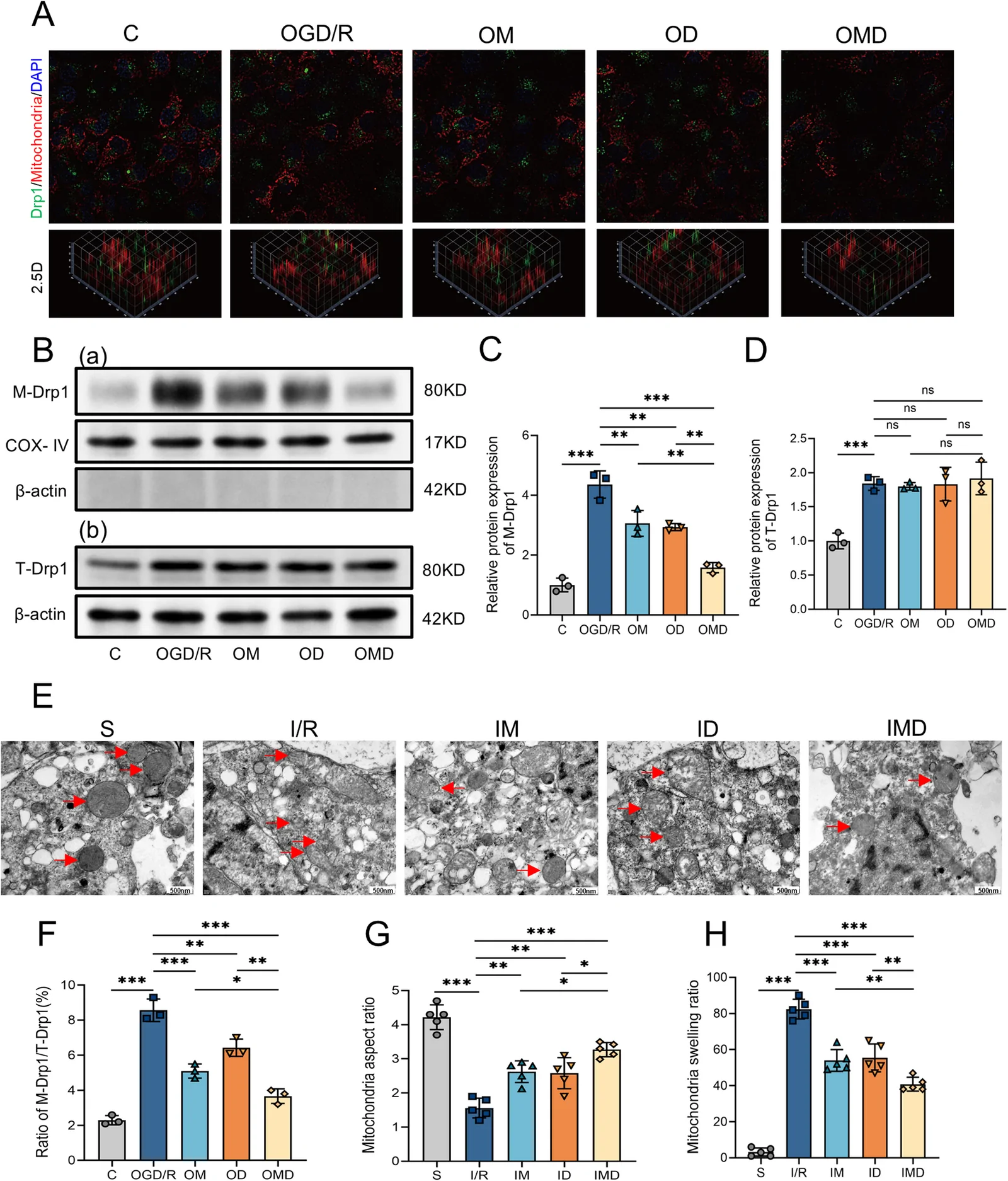
MST1 phosphorylation exacerbates mitochondrial damage through Drp1 activation. (**A**) Representative Immunofluorescence staining examination of Drp1(green), Mitochondria(red), and DAPI (blue) under confocal microscope (63 ×), the co-localization of green and red signals was labeled as M-Drp1. (**B**) (a) Representative Western blot bands showing the protein expression levels of M-Drp1 in BV-2 cells. COX-Ⅳ was used as a loading control for mitochondrial protein. Mitochondrial fraction purity was verified by the absence of β-actin. (b) Representative Western blot bands showing the protein expression levels of T-Drp1 in BV-2 cells. β-actin was used as a loading control. (**C**,**D**) Densitometric quantification of relative M-Drp1 (**C**) and T-Drp1 (**D**) proteins levels normalized to COX- Ⅳ and β-actin, respectively. (*n* = 3). (**E**) Representative TEM images showing the ultrastructure of mitochondria (red arrows) in the ischemic penumbra. Scale bar: 50 μm. (**F**) Quantitative analysis of the Drp1 translocation to mitochondria under confocal microscope. The bar graph represents the ratio of M-Drp1/T-Drp1. (*n* = 3). (**G**,**H**) The bar graphs represent the aspect ratio (**G**) and swelling ratio (**H**) to assess changes in mitochondrial ultrastructure. (*n* = 5). Data are expressed as mean ± SD. Statistical analysis was conducted via one-way ANOVA followed by Tukey’s post hoc test. *p < 0.05, **p < 0.01, ***p < 0.001; ns, no significant, *p*>0.05.

Drp1 is important in mitochondrial fission and becomes activated on the outer membrane of mitochondria following CIRI. Drp1 activation can be assessed by measuring the levels of mitochondria-localized Drp1 (M-Drp1) in the outer mitochondrial membrane^31^. Mitochondria (red) and Drp1 (green) co-localization marked as M-Drp1 in BV-2 cells was examined using immunofluorescence staining combined with confocal microscopy (Fig. 3A). Confocal microscopy showed that the ratio of M-Drp1/T-Drp1 was increased after OGD/R. Intervention with Mdivi-1 decreased the ratio, while suppression of MST1 presented the similar effect on the ratio. In addition, the combined interventions resulted in a greater reduction in the ratio than mono-inhibition (Fig. 3F).

Additionally, we assessed M-Drp1 levels through Western blot after separating mitochondrial and cytoplasmic fractions from BV-2 cells. The purity of the isolated mitochondria was verified by the absence of the cytosolic marker β-actin in the mitochondrial fraction (Fig. B (a)). The Western blot analysis indicated an increase in both M-Drp1 and T-Drp1 expression after reperfusion. Treatment with either the MST1 or the Drp1 inhibitors reduced the M-Drp1 expression levels, with combined inhibition yielding a further decrease effect. However, inhibition of MST1 or/and Drp1 had no influence on the total Drp1 (Fig. B (b)).

To evaluate the extent of mitochondrial fission following CIRI, TEM was used to analyze the ultrastructure of mitochondria within the cortical ischemic penumbra region (Fig. 3E). The findings indicated that mitochondria in S group maintained an intact ultrastructure with well-defined cristae. In comparison, the I/R group displayed marked ultrastructural injury, including reduced matrix density, disrupted cristae, apparently swollen mitochondria, and a substantially decreased aspect ratio. Either using XMU-MP-1 or Mdivi-1 mitigated these pathological changes, partially restoring cristae density, significantly reducing the proportion of swollen mitochondria, and increasing the mitochondrial aspect ratio compared with the I/R group. Notably, a more pronounced structural repair effect was attained with combined inhibitor treatment, and the double-membrane structures as well as internal cristae of mitochondria exhibited higher integrity and clarity (Fig. 3G, H).

### P-MST1 promotes microglia pro-inflammatory activation via activating Drp1

As microglia pro-inflammatory markers, iNOS and CD86 were detected by Western blot assays to evaluate microglia pro-inflammatory activation of BV-2 cells, as well as by immunofluorescence co-localization analysis of Iba-1 with iNOS and CD86 within the ischemic penumbra.

**Fig. 4:**
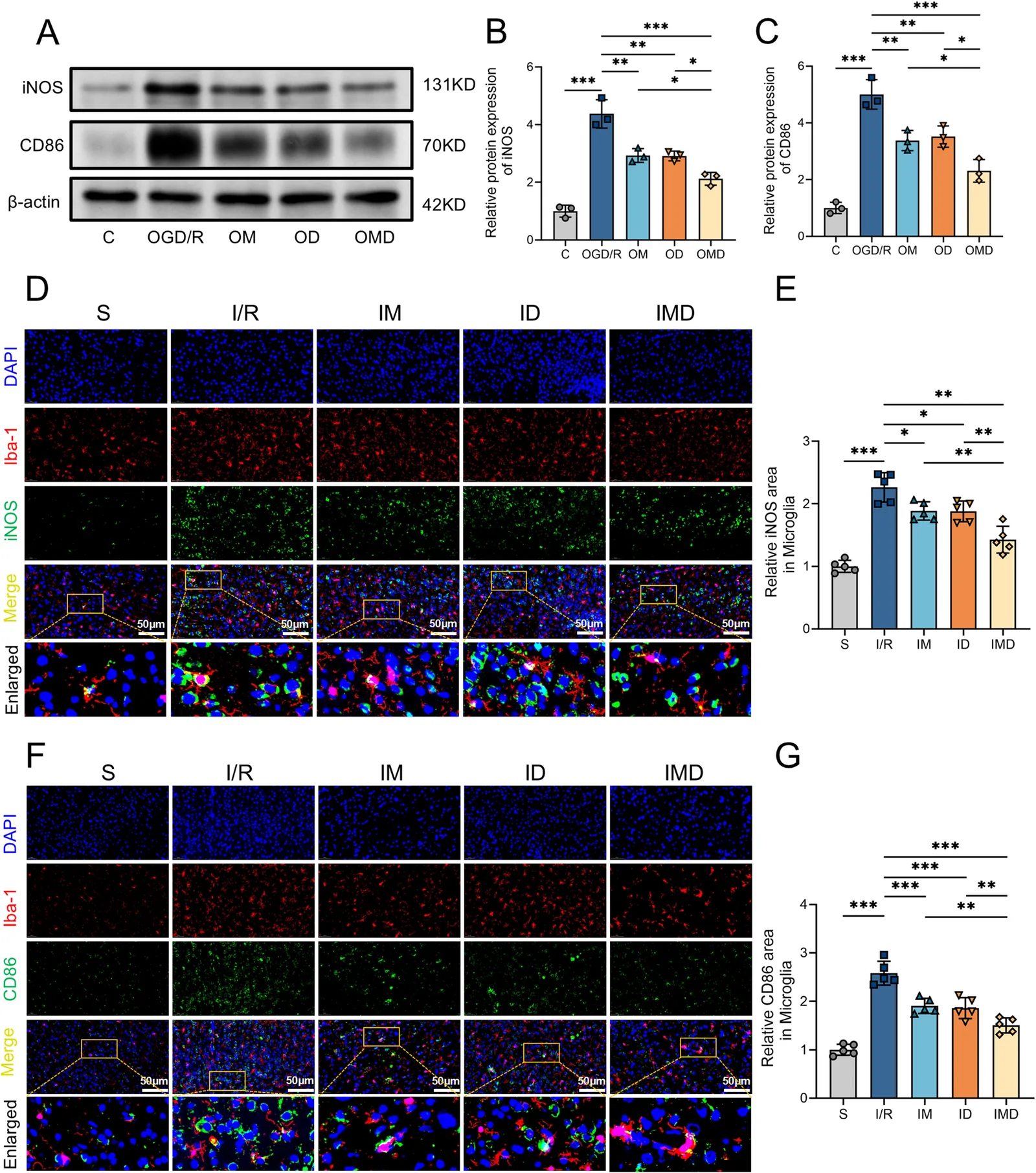
MST1 phosphorylation promotes microglia pro-inflammatory activation via p-Drp1(Ser616) following CIRI. (**A**) Representative Western blot bands showing the protein expression levels of iNOS and CD86 in BV-2 cells. β-actin was used as a loading control. (**B**,**C**) Densitometric quantification of relative iNOS (**B**) and CD86(**C**) proteins levels normalized to β-actin (*n* = 3). (**D**,**F**) Representative immunofluorescence images of iNOS and CD86 (green) co-localization with the microglial marker Iba-1 (red) in the ischemic penumbra. Nuclei were counterstained with DAPI (blue). (**E**,**G**) The bar graphs represent the relative area of co-localization of Iba-1⁺ microglia and iNOS (**E**) or CD86 (**G**) (*n* = 5). Scale bar: 50 μm. Data are expressed as mean ± SD. Statistical analysis was conducted via one-way ANOVA followed by Tukey’s post hoc test. *p < 0.05, **p < 0.01, ***p < 0.001.

In comparison to the C group, treatment with OGD/R significantly elevated the iNOS and CD86 in BV-2 cells. Mono-inhibition of MST1 or Drp1 weaken the iNOS and CD86 expression, and the combined inhibitor regimen results in an additional reduction (Fig. 4A-C). Consistent with outcomes in vitro, immunofluorescence analysis in vivo showed enhanced relative colocalization areas of iNOS or CD86 with Iba-1 in the I/R group. Treatments with either MST1 or Drp1 inhibitor decreased the relative co-localization areas of iNOS or CD86 with Iba-1. Furthermore, the combined inhibitor regimen resulted in an additional reduction of the relative colocalization areas (Fig. 4D-G).

**Fig. 5:**
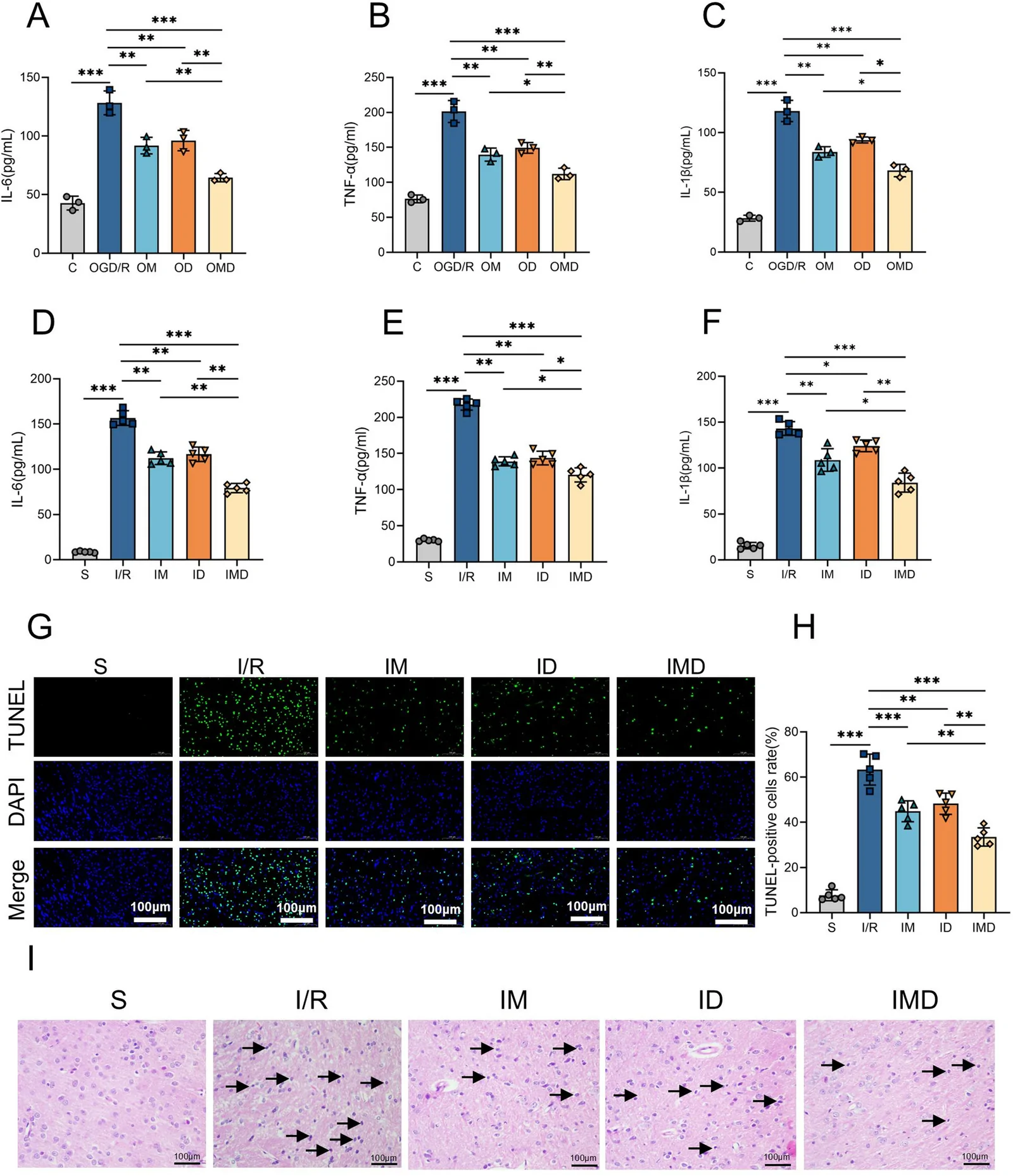
MST1 phosphorylation exacerbates DNA fragmentation and associated pathological alterations via the p-Drp1(Ser616) following CIRI. (**A**–**C**) ELISA results of IL-6 (**A**) or TNF-α (**B**) or IL-1β (**C**) expression levels in BV-2 cell culture medium (*n* = 3). (**D**–**F**) ELISA results of IL-6 (**D**) or TNF-α (**E**) or IL-1β (**F**) expression levels in the ischemic penumbra (*n* = 5). (**G**) Representative images of TUNEL staining in the cerebral ischemic penumbra. Scale bar: 100 μm. (**H**) Quantification of the TUNEL-positive cells rate by TUNEL assay. (*n* = 5). (**I**) Representative images of HE staining in the cerebral ischemic penumbra. Black arrows identify cells displaying morphological abnormalities, such as cellular shrinkage and nuclear pyknosis. Scale bar: 100 μm. (*n* = 5). Data are expressed as mean ± SD. Statistical analysis was conducted via one-way ANOVA followed by Tukey’s post hoc test. *p < 0.05, **p < 0.01, ***p < 0.001.

To evaluate neuroinflammatory levels in BV-2 cells and ischemic penumbral brain tissue, the pro-inflammatory cytokines TNF-α, IL-6, and IL-1β were measured by ELISA. The outcomes indicated that the TNF-α, IL-6, and IL-1β were significantly elevated following I/R and OGD/R. Either MST1 inhibition or Drp1 inhibition markedly decreased the secretion of these cytokines. Notably, combined inhibitor treatment further diminished the expression of these cytokines (Fig. 5A-F).

### P-MST1 enhances DNA fragmentation and morphological changes following CIRI via Drp1 activation

TUNEL immunofluorescence staining was performed to assess DNA fragmentation. The TUNEL-positive signals (green) co-stained with DAPI (blue) were identified as cells with DNA fragmentation (Fig. 5G). Quantitative analysis confirmed that there were few cells with DNA fragmentation in the S group, whereas the I/R group displayed a significant presence of DNA fragmentation within the ischemic penumbra region. In contrast, treatment with either MST1 inhibitor or Drp1 inhibitor markedly reduced the TUNEL-positive rate, with the combined inhibition yielding a more protective effect (Fig. 5H).

Hematoxylin-Eosin (HE) staining was conducted to evaluate changes in the morphology of neural cells following CIRI. In the S group, nerve cells exhibited a well-organized architecture with distinct nuclei and abundant cytoplasm. In contrast, severe histopathological damage, including disordered cell arrangement, nuclear condensation, and cytoplasmic shrinkage, was evident in the I/R group. Notably, treatment with either inhibitor alleviated these morphological abnormalities, whereas combined inhibition more effectively restored the structure of neural cell (Fig. 5I).

### P-MST1 exacerbates neurological damage and cerebral infarct volume via Drp1 activetion

**Fig. 6:**
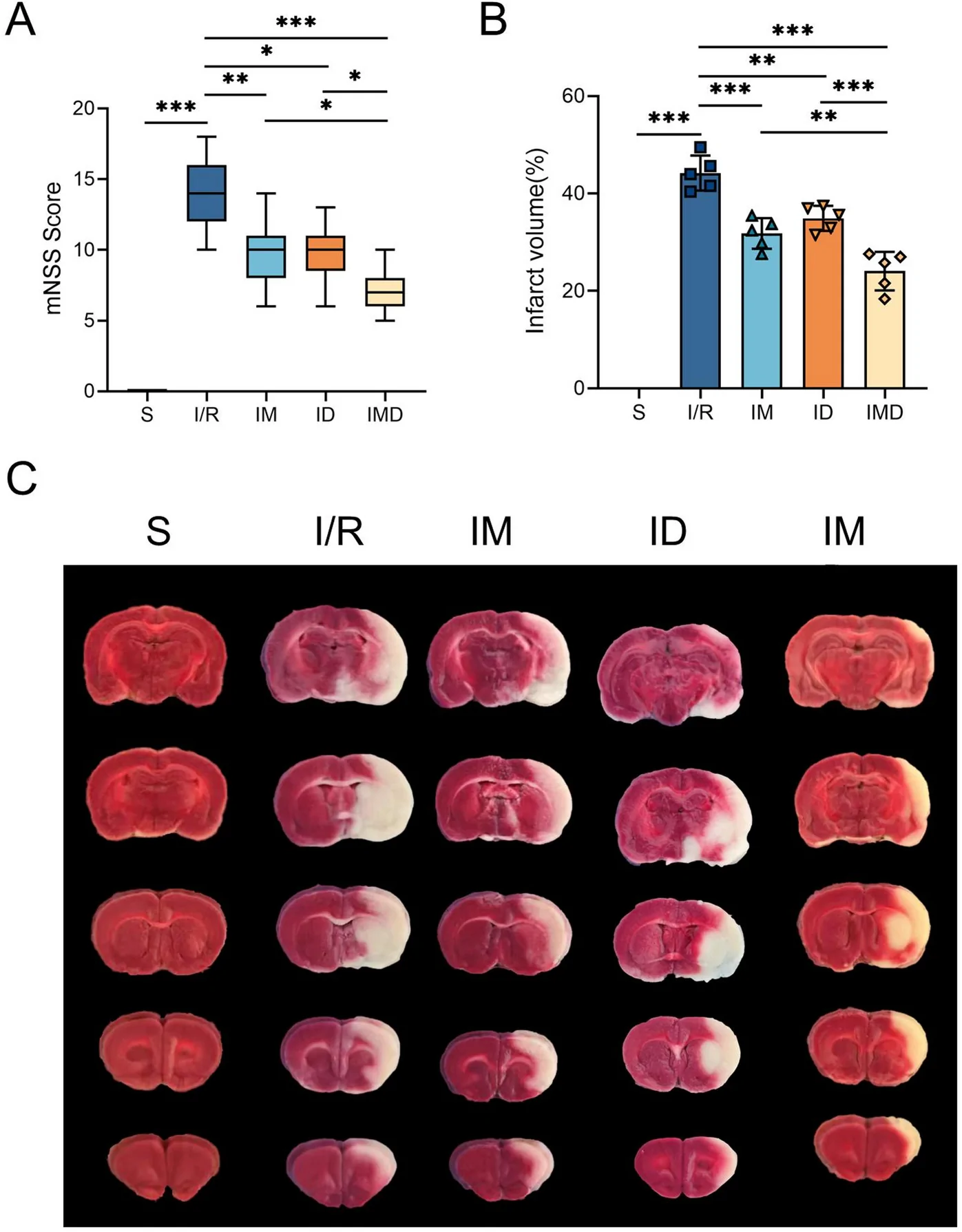
MST1 phosphorylation exacerbates brain tissue damage and neurological deficit via p-Drp1(Ser616) following CIRI. (**A**) Assessment of neurological function (mNSS) in different groups of rats (*n* = 25). Data are expressed as median with IQR. Statistical analysis was conducted via the Kruskal-Wallis test followed by Dunn’s post hoc test. (**B**) Quantification of the percentage of cerebral infarct volume. (**C**) Representative images of TTC-stained brain sections from each group. The non-ischemic area is red and the infarcted area is pale white. (*n* = 5). Data are expressed as mean ± SD. Statistical analysis was conducted via one-way ANOVA followed by Tukey’s post hoc test. *p < 0.05, **p < 0.01, ***p < 0.001.

Neurological function was assessed using the mNSS 24 h after reperfusion in rats, and infarct area was determined through TTC staining. A marked increase in the mNSS was observed in I/R group in comparison to S group. Conversely, treatment inhibitor intervention remarkably diminished the mNSS in the IM, ID, and IMD groups, with the most substantial decrease noted in the IMD group (Fig. 6A). The infarct volume percentage of each group was assessed through TTC staining, in which the red areas represented healthy brain tissue, and the white areas indicated cerebral ischemic tissue (Fig. 6C). Marked differences in infarct volume were observed among the groups following reperfusion. Inhibition of MST1 or Drp1 alone substantially reduced the infarct volume, while combined inhibition further decreased the volume compared with single inhibition (Fig. 6B).

### MST1 enhances microglia pro-inflammatory activation via Drp1 during OGD/R

**Fig. 7:**
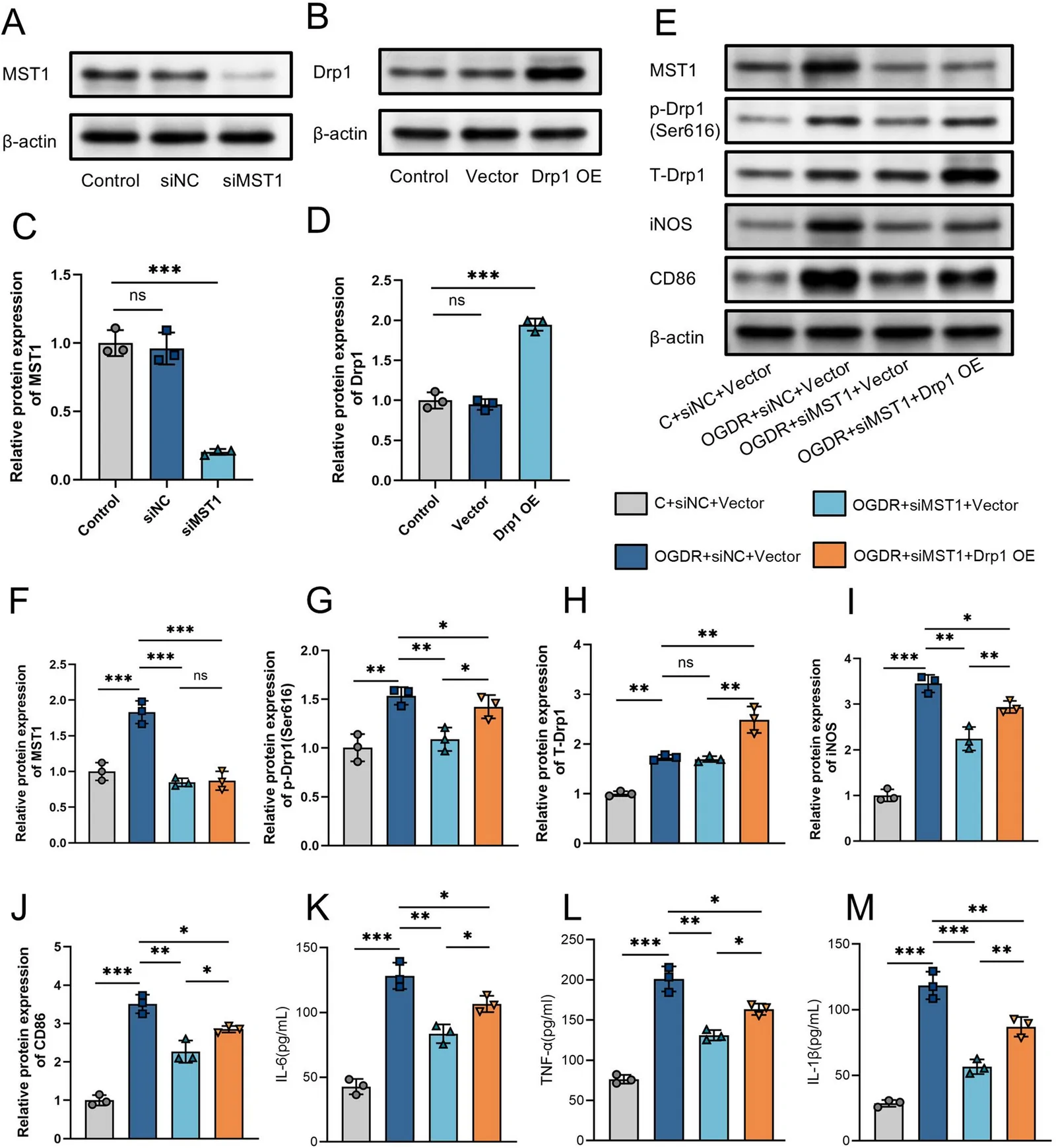
MST1 enhances pro-inflammatory activation p-Drp1(Ser616) during OGD/R. (**A**,**B**) Representative Western blot bands showing the protein expression levels of MST1(**A**) and Drp1(**B**) after the primary microglia transfection. (**C**,**D**) Densitometric quantification of relative MST1 (**C**) and Drp1 (**D**) proteins levels normalized to β-actin (*n* = 3). (**E**) Representative Western blot bands showing the protein expression levels in primary microglia. Drp1 was used as a loading control to p-Drp1(Ser616), β-actin was used as a loading control to other protein. (**F**–**J**) Densitometric quantification of relative proteins levels normalized to Drp1 or β-actin (*n* = 3). (**K**–**M**) ELISA results of IL-6 (**K**) or TNF-α (**L**) or IL-1β (**M**)expression levels in the primary microglia culture medium (*n* = 3). Data are expressed as mean ± SD. Statistical analysis was conducted via one-way ANOVA followed by Tukey’s post hoc test. *p < 0.05, **p < 0.01, ***p < 0.001; *ns*, no significant, *p*>0.05.

Successful knockdown of MST1 and overexpression of Drp1 were verified prior to OGD/R (Fig. 7A-D). As shown in Fig. 7E and F, siMST1 transfection significantly decreased MST1 protein levels compared with the OGD/R + siNC + Vector group, whereas Drp1 overexpression had no effect on MST1 protein expression. Notably, the MST1 knockdown attenuated p-Drp1(Ser616) protein expression without affecting T-Drp1 levels (Fig. 7G-H), indicating that MST1 regulates Drp1 specifically via p-Drp1(Ser616) modification. This result is consistent with the results from the experiments using the MST1 inhibitor. Drp1 overexpression significantly restored p-Drp1(Ser616) protein expression in the OGD/R + siMST1 + Drp1 OE group, thereby reversing the protective effects of MST1 silencing. Furthermore, siMST1 markedly suppressed the protein expression of the pro-inflammatory activation markers iNOS and CD86 under OGD/R conditions. This inhibitory effect was reversed by Drp1 overexpression in the OGD/R + siMST1 + Drp1 OE group (Fig. 7I-J). To evaluate the extent of neuroinflammation after OGD/R in primary microglia, measurements were taken of TNF-α, IL-6 and IL-1β present in the supernatant of cells. Under OGD/R conditions, siMST1 markedly suppressed the secretion of IL-6, TNF-α and IL-1β. This inhibitory effect was reversed by Drp1 overexpression in the OGD/R + siMST1 + Drp1 OE group (Fig. 7K-M).

## Discussion

Stroke is a significant life-threatening health concern worldwide, and more than 80% of cases are ischemic in origin^32^. The primary therapeutic strategy is to restore cerebral blood supply as promptly as possible. However, reperfusion itself can exacerbate neuronal injury through a cascade of mechanisms, including oxidative stress, inflammatory reactions, and programmed cell death. This exacerbation is a critical component of CIRI^33,34^. Neuroinflammation is primarily mediated by microglia and other immune cells, which become activated during CIRI and contribute to secondary neuronal death via sustained inflammatory cascades^35^. Although MST1 or Drp1 has been reported to mediate microglia pro-inflammatory activation in multiple models, the present study further demonstrates that MST1 acts as an upstream regulator of Drp1, and that the MST1/Drp1 signaling axis plays a pivotal role in regulating microglial activation during CIRI. These findings were verified using interventions including inhibition of MST1/Drp1, siMST1, and Drp1 overexpression in both in vitro and in vivo experiments.

MST1 functions as a vital kinase within the Hippo signaling pathway, playing a significant role as a central hub that connects cellular stress, mitochondrial dynamics, immune inflammation, and programmed cell death^36^. Previous research has demonstrated that MST1 is crucial in the development of injuries related to cardiac or renal ischemia/reperfusion, complications arising from diabetes, injuries to the spinal cord, and disorders linked to neuroinflammation^13,21,37^. In a mouse model of CIRI, conditional knockout of MST1 in microglia attenuates neuroinflammation and brain injury by suppressing NF-κB activation^38^. In this research, MST1 and p-MST1 levels were increased following reperfusion, accompanied by mitochondrial ultrastructural damage and nerve injury. In contrast, treatment with the MST1 inhibitor XMU-MP-1 decreased p-MST1 levels and reduced mitochondrial morphological change and neuroinflammation. Notably, XMU-MP-1 is a dual MST1/2 inhibitor. Previous study has shown that MST2 can promote neuronal cell death under oxidative stress conditions, suggesting a potential role in ischemic brain injury^39^. To further clarify the specific function of MST1 independent of MST2, knockdown intervention of MST1 in primary microglia was used. Consistent with the effects of XMU-MP-1, siRNA-mediated MST1 knockdown in primary microglia under OGD/R conditions reduced p-Drp1(Ser616) and attenuated pro-inflammatory activation, further supporting a role for MST1 in regulating microglia pro-inflammatory activation. These results suggest that upregulation and activation of MST1 may be involved in the mechanisms driving mitochondrial morphological change and neuroinflammation.

Drp1 is important in the mitochondrial fission process, with its phosphorylation facilitating excessive fission of mitochondria during CIRI^23,40^. Under stress conditions such as CIRI, Drp1 is activated and translocated from the cytosol to the outer mitochondrial membrane, where it interacts with mitochondrial fission factor (MFF) and fission protein 1 (Fis1). The process induces excessive mitochondrial fission by hydrolyzing GTP to generate energy^41^. The modulation of Drp1 activation is influenced by several post-translational modifications, with phosphorylation being particularly vital. Two phosphorylation modifications of Drp1 are identified: p-Drp1(Ser616) promotes its activation and mitochondrial translocation, whereas p-Drp1(Ser637) inhibits its translocation^42,43^. In this research, both in vivo and in vitro models showed heightened expression of MST1 and p-MST1, which was paralleled by elevated levels of p-Drp1(Ser616). In the in vivo model, inhibition of MST1 alone reduced p-Drp1(Ser616) and mitochondrial Drp1 abundance to a degree comparable to Drp1 inhibition alone, with the combined inhibition producing the strongest effect. In the in vitro model, siMST1 transfection significantly decreased MST1 and p-Drp1(Ser616) protein levels, whereas Drp1 overexpression restored p-Drp1(Ser616) levels without affecting MST1 expression. This observation is consistent with previous report in renal ischemia-reperfusion model^44^, where p-MST1 enhances p-Drp1(Ser616), thereby exacerbating mitochondrial fission. Notably, neither single nor combined inhibitor treatments influenced T-Drp1 or p-Drp1(Ser637) levels. Similarly, in the OGD/R model of primary microglia, MST1 knockdown specifically attenuated p-Drp1(Ser616) protein expression without altering T-Drp1 levels. These results suggest that inhibiting MST1 could lessen excessive mitochondrial fission by decreasing p-Drp1(Ser616), instead of affecting T-Drp1 or p-Drp1(Ser637) following reperfusion or reoxygenation.

In the central nervous system, microglia are the innate immune cells, demonstrating swift reactions to CIRI and possessing the ability to trigger strong neuroinflammatory processes^45,46^. Microglia exist as a continuum of dynamic functional states, ranging from homeostatic microglia to reactive/responding microglia and various context-dependent functional states^47^. During the acute phase of CIRI at 24 h after reperfusion, proinflammatory and neurotoxic microglial subcluster is predominant characterized by activation of TNF-α and NF-κB signaling pathways^48^. CD86 and iNOS surface markers of microglia activation from a homeostatic state into pro-inflammatory state^49,50^. CD86 is expressed by activated microglia and serves as a marker of microglia pro-inflammatory activation^51^. During ischemic stroke, activated iNOS-positive microglia trigger inflammatory cascades and induce neuronal damage^52^. IL-1β, IL-6, and TNF-α are key pro-inflammatory cytokines secreted by activated microglia^53^. Accordingly, in the present study, 24 h after reperfusion or reoxygenation was set as the experimental detection time points. Increased expressions of the iNOS and CD86, along with a rise of TNF-α, IL-6 and IL-1β, suggest a heightened microglia pro-inflammatory activation level following reperfusion or reoxygenation.

Excessive mitochondrial fission promotes microglia pro-inflammatory activation. Drp1 mediated mitochondrial damage triggers microglia pro-inflammatory activation, which contributes critically to neuroinflammation in various CNS disorders^54^. In the research of the Parkinson’s disease model, Drp1-mediated mitochondrial fission induces a pro-inflammatory response in microglia of G2019S TG mice brains^55^. Pharmacological inhibition of Drp1 using Mdivi-1 or siRNA-mediated Drp1 knockdown suppressed mitochondrial fragmentation, reduced the levels of inflammatory factors including TNF-α, IL-6, and IL-1β, and alleviated neuronal damage^26^. Katoh et al.^56^ demonstrated that activated Drp1 promotes mitochondrial fission and drives pro-inflammatory activation in LPS stimulated primary microglia and in a mouse demyelinating model. In this study, inhibition of MST1 or/and Drp1 resulted in attenuated excessive mitochondrial damage, and subsequently suppress microglia pro-inflammatory activation and inflammatory. However, Mdivi-1 has been shown to influence mitochondrial function independently of Drp1. Mdivi-1 reversibly inhibits mitochondrial complex I-dependent O_2_ consumption and reverse electron transfer-mediated reactive oxygen species (ROS) production at 50 µM concentrations^57^, and these actions may contribute to the anti-inflammatory and protective effects of mdivi-1. In our experiments of primary microglia, Drp1 overexpression was used to further validate the role of Drp1 in microglial activation. siMST1 significantly suppressed the expression of the microglial activation surface markers iNOS and CD86 under OGD/R conditions, and this inhibitory effect was reversed by Drp1 overexpression. Collectively, these findings indicate that p-MST1 drives microglia pro-inflammatory activation via Drp1-mediated excessive mitochondrial fission during CIRI.

Overall, inhibition of p-MST1 impacts the levels of phosphorylation Drp1(Ser616), consequently affecting downstream processes including excessive mitochondrial fission and the microglia pro-inflammatory activation. Conversely, Drp1 inhibition exclusively influences p-Drp1(Ser616) and its subsequent effectors, without altering MST1 or its phosphorylation. Consistent with these findings, in the OGD/R model of primary microglia, siMST1 attenuated p-Drp1(Ser616) expression and decreased the expression of iNOS and CD86. Conversely, Drp1 overexpression restored p-Drp1(Ser616) levels and the expression of iNOS and CD86, without altering MST1 expression. These observations confirm that Drp1 serves as one of the downstream targets of MST1.

Nevertheless, this study has limitations. First, the present study did not investigate the multiple time points to evaluate the temporal dynamics of microglial activation states. Second, the impact of MST1/Drp1 axis on other microglial functional states was not explored. Finally, rescue experiments involving elevation of p-Drp1(Ser616) levels via site-directed mutagenesis combined with MST1 inhibition could be performed to further confirm the role of the MST1/Drp1 axis in regulating microglia pro-inflammatory activation.

In conclusion, this research highlighted the regulatory function of the MST1/Drp1 axis during CIRI. Activation of MST1 following reperfusion promotes Drp1 phosphorylation at Ser616, which triggers excessive mitochondrial ultrastructural damage, subsequently drives microglia pro-inflammatory activation, and ultimately contributes to the pathogenesis of CIRI. Targeting the microglial MST1/Drp1 axis may constitute a promising therapeutic approach to attenuate neuroinflammation and confer neuroprotection in CIRI.

## Supplementary Information

Below is the link to the electronic supplementary material.


Supplementary Material 1


## Data Availability

The datasets generated and analyzed during the current study are available from the corresponding author on reasonable request.
