# Supplementary material for: MST1/Drp1 axis mediates microglia pro-inflammatory activation following cerebral ischemia-reperfusion injury
Source: Sci Rep. 2026 Jun 13;16:27062. doi: 10.1038/s41598-026-57741-6 (PMC13521907; doi:10.1038/s41598-026-57741-6)
Supplement: Supplementary file 1 [file 41598_2026_57741_MOESM1_ESM.docx]

MST1(56KD), p-MST1(56KD), β-actin(42KD)

p-Drp1(Ser616) (80KD), β-actin(42KD)

p-Drp1(Ser637) (80KD), β-actin(42KD)

M-Drp1(80KD), COX-IV(17KD), β-actin(42KD)

T-Drp1(80KD), β-actin(42KD)

iNOS(131KD), CD86(70KD), β-actin(42KD)

P
